# Advancements in extracellular vesicles biomanufacturing: a comprehensive overview of large-scale production and clinical research

**DOI:** 10.3389/fbioe.2025.1487627

**Published:** 2025-02-19

**Authors:** Ziqian Li, Junyu Yan, Xiang Li, Hui Chen, Chen Lin, Yuhang Zhang, Tian Gao, Yabo Zhang, Yue Shu, Shuyuan Pan, Yuntao Zhang

**Affiliations:** ^1^ Beijing Institute of Biological Products Company Limited, Beijing, China; ^2^ China National Biotec Group Company Limited, Beijing, China

**Keywords:** extracellular vesicles, biomarkers, drug delivery, large-scale production, characterization

## Abstract

Extracellular vesicles (EVs) are nano-sized, membranous structures secreted by cells into the extracellular space, have attracted considerable attention in the field of biosciences for their role in intercellular communication in various physiological and pathological processes. Their ubiquitous presence in bodily fluids and cell-specific characteristics make them promising candidates as biomarkers. Additionally, their ability to transport biological therapeutics across different biological barriers to specific target cells underscores their significant translational potential for diagnostic and therapeutic purposes. Significant progress has been achieved in the translation of EVs research to clinical applications, however, challenges persist in the large-scale production of EVs, particularly in the areas of scalable manufacturing, efficient isolation methods, drug loading techniques, and advanced characterization technology. This review critically examines the complex processes involved in EVs biogenesis and explores recent developments in large-scale EVs production. By synthesizing knowledge from these fields, this review aims to provide a holistic perspective on the evolving landscape of EVs research and its applications, underscoring both the accomplishments and the obstacles that lie ahead in fully realizing the potential of EVs in biomedicine.

## 1 Introduction

Extracellular vesicles (EVs) are small particles composed of proteins, lipids, and nucleic acids, secreted by various types of cell lineages (Shu et al., 2021; Wu et al., 2021; Wright et al., 2023; Arellano et al., 2024; Liang et al., 2024). They cannot replicate on their own and are enclosed by a lipid bilayer. EVs carry information from their cell of origin, such as proteins, mRNAs, microRNAs, and non-coding RNAs (Deville et al., 2021; Kronstadt et al., 2023b; Susa et al., 2023; Qian et al., 2024). EVs encompass multiple subtypes, including exosomes, microvesicles, apoptotic bodies, and others (Ju et al., 2023; Wen et al., 2023). Due to the current limitations in separation technologies that make it challenging to enrich EVs produced by different mechanisms and to characterize the corresponding EV subpopulations, most studies continue to use the term “exosome” to represent a broad population of EVs (Welsh et al., 2024). In the groundbreaking year of 1981, Trams and associates utilized transmission electron microscopy to visually demarcate a distinct class of vesicles, measuring 40–1,000 nm in breadth, marking a seminal juncture in deciphering the heterogeneity of extracellular vesicles (EVs) (Trams et al., 1981). Subsequently, Johnstone’s pioneering work in the late 1980s elucidated the essential presence of membranous vesicles in reticulocyte maturation, which could be isolated through ultracentrifugation and were subsequently termed exosomes (Johnstone et al., 1987). Initially misconceived as mere metabolic debris, EVs have since been redefined as complex mediators of intercellular communication.

**FIGURE 1:**
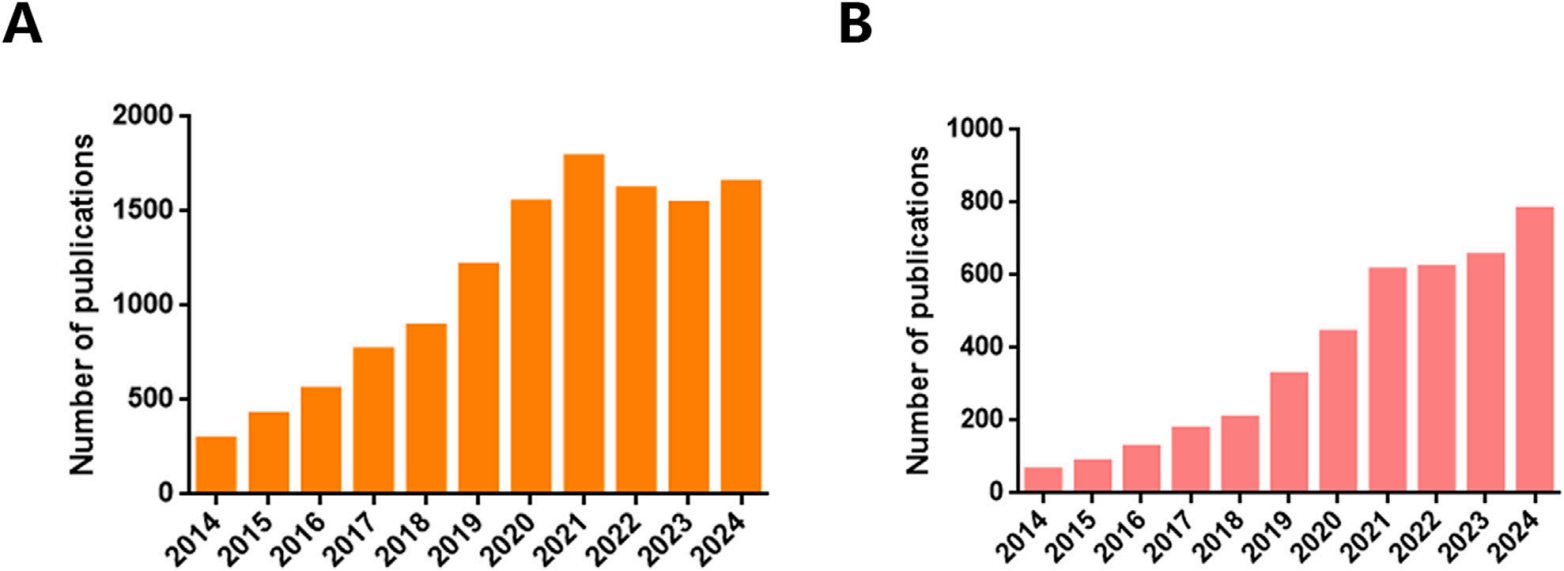
Number of publications. The number of Extracellular vesicles publications between 2014 and 2024 searched by **(A)** “biomarkers” and **(B)** “drug delivery” keyword, according to PubMed.

In contemporary biomedical research, EVs are highly regarded for their roles as crucial intermediaries in cellular communication, regulators of systemic homeostasis, and potential vehicles for paracrine signaling molecules, thereby stimulating intensified investigation into their therapeutic potential, particularly in the fields of drug delivery and biomarker discovery (Zhang et al., 2019; Kalluri and LeBleu, 2020; Patil et al., 2020; Kucuk et al., 2021). Comprehensive datasets cataloging EVs identification, functional annotations, and protein interactomes are meticulously maintained in repositories such as ExoCarta (Author anonymous, 2024), EV-TRACK (Consortium et al., 2017), Vesiclepedia (Chitti et al., 2024), exRNA Atlas (Murillo et al., 2019), and EVpedia (Kim et al., 2015). These repositories highlight the ubiquitous presence of EVs across diverse life forms, ranging from archaea and bacteria to higher eukaryotes, including humans. The intrinsic capabilities of EVs to traverse physiological barriers, evade immune detection, and counteract efflux-mediated drug resistance underscore their appeal as precision-engineered nanocarriers for targeted therapy (Patil et al., 2020). Their biocompatibility, minimal cytotoxic effects, and potential for tailored cell-specific modifications further enhance their status as innovative biological vectors, a theme recurrent in scientific discourse (Patil et al., 2020). According to inquiries in the NCBI PubMed database, as of November 2024, the past 5 years have witnessed an exponential increase in publications concerning EVs as biomarkers, totaling 8,145 articles. This represents a significant 211% increase compared to the 2014–2019 period (Figure 1A). Comparatively, although fewer in number, studies concentrating on EVs for drug delivery (3,113) publications within the same period) have investigated the transport of small molecules (Figure 1B), macromolecular complexes, and nucleic acids. This reflects a burgeoning research interest and the expanding potential of EVs applications in therapeutics and diagnostics. In response to the need for standardized practices and analytical rigor, the International Society for Extracellular Vesicles (ISEV) issued the Minimal Information for Studies of Extracellular Vesicles (MISEV) guidelines in 2014, which were subsequently revised in 2023, reinforcing the methodological bedrock of EVs research (Smith et al., 1991).

Despite these advancements, the practical application of EVs-based therapies faces numerous challenges, particularly in the areas of scalable mass production, refinement of isolation techniques, and strategic loading methodologies to ensure clinical efficacy. These obstacles necessitate ongoing and rigorous research efforts. Concurrently, clinical investigations are meticulously examining the safety and therapeutic efficacy of EVs in both diagnostic and therapeutic contexts, underscoring the dual potential and complexities of utilizing these nanocarriers for precision medicine.

## 2 Extracellular vesicle biogenesis

### 2.1 Extracellular vesicle biogenesis pathways

**FIGURE 2:**
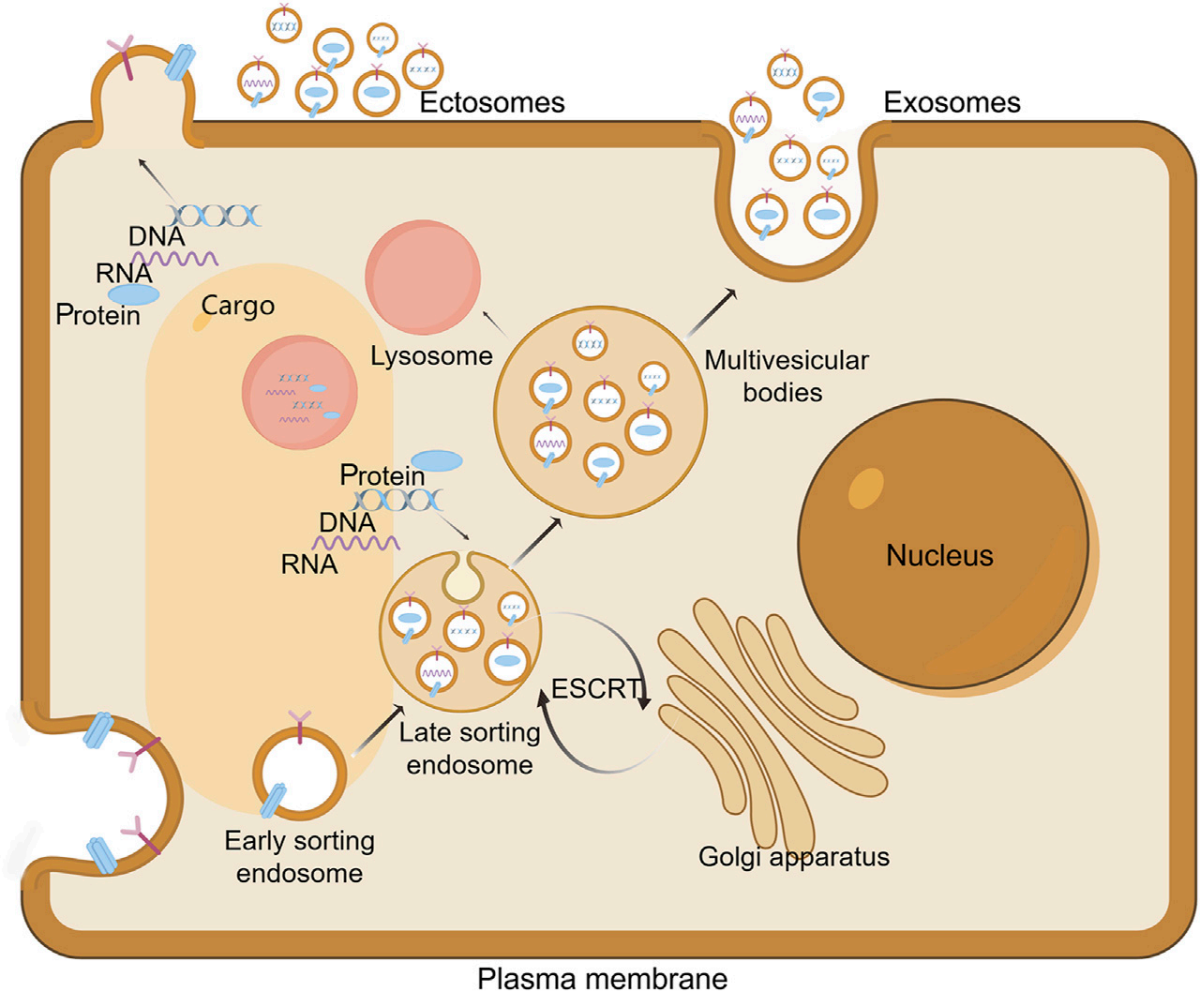
Extracellular vesicles Biogenesis. The biogenesis of exosomes commences with the inward budding of the cellular membrane, resulting in the formation of cup-shaped structures that subsequently develop into early endosomes. Within these compartments, further invagination of the endosomal membrane occurs, encapsulating cytoplasmic contents such as DNA, RNA, and proteins. This process leads to the budding off of intraluminal vesicles (ILVs), thereby forming late endosomes. The maturation of these endosomes is facilitated by contributions from the Golgi apparatus, which enriches the endosomal content. Subsequently, the late endosomes mature into multivesicular bodies (MVBs). Ultimately, MVBs undergo fusion with the plasma membrane, resulting in the extracellular release of ILVs as exosomes. Alternatively, some MVBs may fuse with lysosomes, thereby targeting their contents for degradation. On the other hand, ectosomes arise from outward protrusions of plasma membrane that are excised and shed into the extracellular space. Draw by FigDraw.

The biogenesis of extracellular vesicles (EVs) is currently understood to revolve around two primary mechanisms: the endosomal pathway and the plasma membrane-mediated route (Figure 2) (Yu et al., 2023). The endosomal pathway is the more prominent of the two, initiated by the budding of the plasma membrane to form early endosomes (Zhang et al., 2019). Within these endosomes, intraluminal vesicles (ILVs) subsequently bud, leading to the formation of multivesicular bodies (MVBs). These MVBs eventually fuse with the plasma membrane to release exosomes (Han et al., 2019). This paradigm, deeply entrenched in the investigation of transferrin receptor dynamics during reticulocyte differentiation, has been extensively utilized to elucidate the mechanisms of exosome biogenesis (Willson, 2020). Concurrently, an increasing body of evidence highlights a direct plasma membrane-originating pathway, wherein etcosomes are generated through budding without the involvement of multivesicular body (MVB) intermediates (Xie et al., 2022).

Specifically, in the context of human CD4^+^ T lymphocytes, exosome secretion occurs directly from specialized “endosome-like microdomains” within the plasma membrane (Baietti et al., 2012). These microdomains are characterized by a high concentration of key exosomal markers, such as CD63 and CD81, and an assortment of endosomal proteins (van Niel et al., 2011). Utilizing targeted labeling of CD63, CD81, and CD9 in both plasma membrane and endosomal compartments, research has identified the plasma membrane of human embryonic renal cells and murine NIH3T3 fibroblasts as primary sites for exosome biogenesis (Mathieu et al., 2021). These vesicles, which are indistinguishable in size, molecular markers, proteomic profiles, and lipid composition from their multivesicular body (MVB)-originated counterparts, support the hypothesis that direct plasma membrane budding is a plausible mechanism for exosome production (Mathieu et al., 2021).

Notably, certain cellular contexts deviate from the traditional multivesicular body (MVB) and plasma membrane pathways. Macrophages serve as a notable example of this deviation, with intracellular plasma membrane-connected compartments (IPMCs) proposed as alternative biogenetic niches for exosomes (Nkwe et al., 2016). Unlike MVBs, IPMCs retain a neck connection to the plasma membrane, facilitating the free passage of small molecules to the extracellular environment (Bebelman et al., 2020). In the context of HIV-infected macrophages, IPMCs have been identified as significant sites for viral particle egress. Perturbations that disrupt membranous continuity facilitate the release of CD81/CD9-bearing exosomes along with other membranous components (Nkwe et al., 2016). This mechanism, however, seems to be a unique feature of macrophage biology and is not widely observed across different cell types.

Collectively, these findings highlight the complex nature of extracellular vesicle (EV) biogenesis, which varies among cell types, with some exhibiting unique or predominant biogenetic pathways. This heterogeneity adds complexity to our understanding of EVs as sophisticated mediators in cellular communication and function.

### 2.2 Extracellular vesicles biogenesis mechanisms

Current research on EVs biogenesis predominantly focuses on the endosomal pathway, wherein nascent endosomes progressively mature into MVBs through mechanisms that involve either endosomal sorting complex required for transport (ESCRT) -dependent or ESCRT-independent processes (Wei et al., 2021). The ESCRT machinery represents a sophisticated ensemble of protein complexes, including heterodimeric assemblies such as ESCRT-0, which consists of HRS (hepatocyte growth factor-regulated tyrosine kinase substrate) and STAM (signal-transducing adapter molecule); tetrameric complexes like ESCRT-I, composed of TSG101, VPS28, VPS37, and MVB12; ESCRT-II, which is formed by EAP45, EAP30, and two EAP20 subunits; ESCRT-III, constituted by CHMP proteins (charged multivesicular body proteins) including CHMP2-6; the ATPase VPS4, which is involved in vacuolar protein sorting; and Bro1 domain proteins, such as ALIX and HD-PTP (Christ et al., 2017; Cada et al., 2022; Olmos, 2022).

**FIGURE 3:**
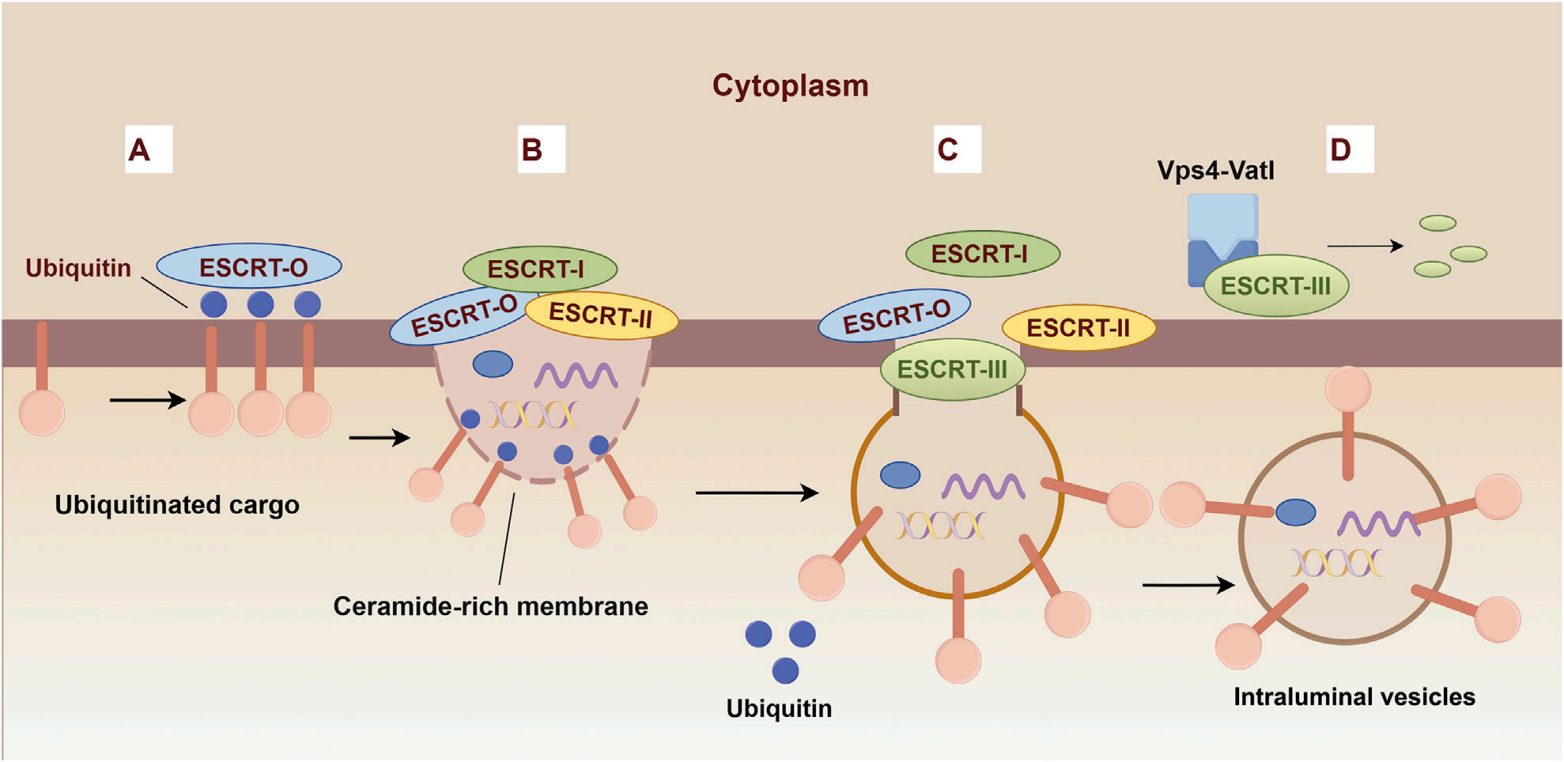
Biogenesis mechanisms of ESCRT-dependent Extracellular vesicles. **(A)** The ESCRT-0 subunits recognize and bind to ubiquitinated cargo proteins, thereby initiating the vesicle formation pathway. **(B)** ESCRT-0 recruits ESCRT-I and ESCRT-II to promote inward budding. **(C)** The ESCRT-III complex assembles at the site of the budding membrane and facilitates the constriction and scission of the bud neck, leading to the formation of intraluminal vesicles (ILVs). **(D)** The ESCRT-III complex is disassembled by ATP hydrolysis catalyzed by Vps4.

The ESCRT-dependent MVB biogenesis protocol comprises a series of four intricately coordinated events: aggregation of selectively ubiquitinated cargo proteins, membrane invagination, vesicle budding, and subsequent vesicle neck scission (Figure 3). Each step is meticulously orchestrated by the sequential deployment of ESCRT complexes (Williams and Urbe, 2007; Henne et al., 2011). The process of cargo clustering is initiated by the HRS-STAM complex, which utilizes the HRS ubiquitin-interacting motif (UIM) to tether ubiquitinated cargo to the endosomal membrane, thereby constructing a protein scaffold for sequestration (Takahashi et al., 2015). The components of ESCRT-I and ESCRT-II, including the VPS27, MVB12 C-termini, and GLUE domain, respectively, play a crucial role in cargo aggregation (Katzmann et al., 2003; Teo et al., 2006; Boura and Hurley, 2012). Sequentially, the HRSPASP motifs in HRS-STAM recruit TSG101, thereby assembling the ESCRT-0 and ESCRT-I complexes, which direct aggregated cargo towards nascent vesicles, transporting ubiquitinated and PT/SAP-bearing proteins into the lumen (Wang et al., 2021). The C-terminus of VPS28 in ESCRT-I orchestrates the recruitment of ESCRT-II, which is essential for membrane deformation, vesicle packaging, and the recruitment of the ubiquitin-binding domain of ESCRT-0 to the budding site (Dionisio-Vicuna et al., 2018). ESCRT-III, subsequently recruited by ESCRT-II or ALIX, facilitates vesicle encapsulation, while VPS4 catalyzes membrane fission and the recycling of factors, culminating in MVB maturation (McCullough et al., 2018).

Furthermore, the Syndecan-Syntenin-ALIX axis has emerged as a pivotal regulator, governing an estimated 50% of exosome biogenesis (Ghossoub et al., 2014). This pathway is mediated through the interaction of membrane-tethered Syndecan with its cytosolic receptor Syntenin, utilizing a LY motif to engage ALIX (Baietti et al., 2012). The recruitment of ALIX to intravesicular sites facilitates the biogenesis of ILVs, establishing a prerequisite for the formation of CD63-positive ILVs(Bissig and Gruenberg, 2014). This process reveals an additional regulatory layer in the complex dynamics of exosome production.

Although the ESCRT machinery is crucial, it does not exclusively govern exosome biogenesis. Numerous studies have demonstrated only modest reductions in exosome production following ESCRT inhibition (Matusek et al., 2019; Addi et al., 2020; Olmos, 2022). The aggregation of evidence for alternative pathways supports their classification as ESCRT-independent mechanisms. Foremost among these alternatives, the ceramide (neutral sphingomyelinase, nSMase) pathway has garnered substantial scientific interest (Guo et al., 2015). Investigative studies on the trafficking dynamics of proteolipid protein (PLP) in Oli-neu cells have revealed PLP’s ESCRT-independent entry into endosomes. Furthermore, the significant reduction in exosome and PLP release following treatment with nSMase inhibitors, such as GW4869, suggests that ceramide promotes ILVs formation for exosomal release rather than lysosomal targeting (Guo et al., 2015).

This concept has been corroborated by numerous additional studies, such as those examining macrophage-to-dendritic cell antigen presentation and miRNA conveyance, processes that are partially dependent on the ceramide-mediated pathway. Notably, within the ceramide signaling cascade, the inhibition of sphingosine-1-phosphate (S1P), a downstream metabolite, impedes ceramide-induced CD63-positive exosome release without reducing the overall exosome output. This observation underscores S1P’s pivotal role in regulating the compartmentalization of ILVs cargo (Han, 2022).

Expanding upon the ceramide paradigm, CD63 has been implicated in the non-ESCRT-mediated sorting of premelanosome protein (PMEL) in human melanoma cells, where suppression of CD63 redirects PMEL towards ESCRT-mediated degradation (Edgar et al., 2014). In HEK293 cells, CD82 and CD9 are instrumental in facilitating the exosomal export of vesicles containing β-catenin. Additionally, in pancreatic carcinoma, tetraspanin 8 plays a crucial role in the selective recruitment of specific proteins and mRNA species into the exosomal cargo (Chairoungdua et al., 2010). These examples collectively illustrate ESCRT-independent mechanisms of exosome biogenesis.

Importantly, the inhibition of any single biogenesis pathway does not completely eliminate exosome production, suggesting that cells possess multiple mechanisms for MVBs formation. Furthermore, MVBs can contain a heterogeneous mix of ILV soriginaing from different pathways, with some ILVs targeted for lysosomal degradation and others for fusion with the plasma membrane, leading to exosome release into the extracellular environment (Bebelman et al., 2020). This variability highlights the complex nature of MVB populations within a single cell type and the intricate dynamics underlying exosome biogenesis.

## 3 Large-scale production of extracellular vesicles

### 3.1 Biofabrication of extracellular vesicles

The biofabrication of EVs requires a comprehensive, multidimensional approach that includes the selection of appropriate cell lines, optimization of bioreactor processes, and induction of EV secretion (Pan et al., 2023).

EVs derived from various cell lineages display a wide array of homing properties, which are intrinsically linked to the unique characteristics of their membrane architecture and the compositional diversity of their molecular cargo (Park et al., 2019; Vazquez-Rios et al., 2019; Ganesh et al., 2022). Notably, EVs originating from tumor cells often exhibit a pronounced tendency to home to tumor tissues and associated lymphatic structures (Vazquez-Rios et al., 2019; Qiao et al., 2020); EVs derived from mesenchymal stem cells (MSCs) exhibit a preferential targeting of injured tissues, underscoring their regenerative homing bias (Nooshabadi et al., 2018; Sun et al., 2021; Jin et al., 2023). During the selection of cell lines, it is crucial to conduct a comprehensive evaluation that includes secretory activity, phenotypic stability, and inherent biosafety profiles to ensure a robust foundation for subsequent bioprocessing efforts (Qu et al., 2023). Cell lines predominantly employed for large-scale EVs production are those characterized by high-efficiency molecular expression profiles, notably including immunological, stem, and neoplastic cell types. Representative examples encompass the CHO cell line, HEK293 cell line, MSCs, and a variety of human tumor cell lines (Table 1) (Guo et al., 2019; Chen et al., 2023; Gao et al., 2024; Wysor and Marcus, 2024; Zhang et al., 2024b; Zhou et al., 2024). All abbreviations and related definitions can be found in the Supplementary Table S1. Recent advancements in the engineering of EVs have significantly broadened their application spectrum, augmenting their potential in targeted drug delivery, regenerative medicine, and diagnostic methodologies (Heidari et al., 2020; Liang et al., 2020; Guo et al., 2022; Yuan et al., 2023).

**TABLE 1:** EVs biofabrication and isolation methods in different bioreactors.

Cell lines	Cell culture method	Isolation method	Treatment targets	References
USCs	Hollow fiber bioreactors	Differential centrifugation, Ultracentrifugation	Nonobstructive azoospermia	Deng et al. (2019)
SHEDs	3D cell culture bioreactors	Differential centrifugation, Ultracentrifugation	Parkinson’s disease	Jarmalaviciute et al. (2015)
MSCs	Hollow fiber bioreactors	Ultracentrifugation	Pancreatic cancer	Mendt et al. (2018)
MSCs	3D cell culture bioreactors	Ultracentrifugation	Osteoarthritis	Duan et al. (2022)
MSCs	Hollow fiber bioreactors	Ultracentrifugation	Cartilage injury or defect	Yan and Wu (2020)
MSCs	Hollow fiber bioreactors	Differential centrifugation	Acute renal injury	Cao et al. (2020)
MSCs	Hollow fiber bioreactors	Ultracentrifugation	Hematopoietic acute radiation syndrome	Kink et al. (2024)
MSCs	3D cell culture bioreactors	Differential centrifugation, Ultracentrifugation	Lung inflammation	Zhang et al. (2024a)
MSCs	3D cell culture bioreactors	Differential centrifugation, Ultracentrifugation	Diabetes	Kronstadt et al. (2023a)
MSCs	3D cell culture bioreactors	Ultracentrifugation	Traumatic Brain Injury	Jalilian et al. (2022)
MSCs	Hollow fiber bioreactors	Differential centrifugation, Ultracentrifugation,SEC	Immunoregulation	Jakl et al. (2023)
MPCs	3D cell culture bioreactors	Ultracentrifugation	Damaged articular cartilage	Phelps et al. (2022)
Cancer Cells (MEC-1)	CELLine bioreactors	Differential centrifugation, Density gradient centrifugation	Leukemia	Wierz et al. (2019)
MEC-1	CELLine bioreactors	Differential centrifugation, Density gradient centrifugation	Chronic lymphocytic leukemi	Elgamal et al. (2020)
h-GECs, h-PODs	3D cell culture bioreactors	Differential centrifugation, Ultracentrifugation	Glomerular injury	Bellucci et al. (2021)
HEK293	Hollow fiber bioreactors	Differential centrifugation	Breast cancer	Watson et al. (2016)
HEK293	Hollow fiber bioreactors	Ultracentrifugation, SEC, Ultrafitration	Immunotherapy	Watson et al. (2018)
HEK293	3D cell culture bioreactors	Ultracentrifugation, Ultrafitration, SEC	Wound healing	Kronstadt et al. (2023b)
HEK293	3D cell culture bioreactors	Ultracentrifugation	Neuroendocrine carcinoma	Si et al. (2020)
HDMECs	3D cell culture bioreactors	Differential centrifugation	Atherosclerosis	Patel et al. (2019)
Cancer Cells (T24)	CELLine bioreactors	Ultracentrifugation	Bladder cancer	Jeppesen et al. (2014)
Cancer Cells (HELA, U87), HEK293	CELLine bioreactors	Affinity chromatography	Invasive cancer	Griffiths et al. (2019)
Cancer Cells (SH-SY5Y)	3D cell culture bioreactors	Ultracentrifugation	Frontal and temporal lobe degeneration	Tunesi et al. (2016)
Cancer Cells (BT-474)	CELLine bioreactors	Differential centrifugation, Ultracentrifugation, SEC	Breast cancer	Hisey et al. (2020)

Bioreactors are central to cell cultivation, with their design and operational parameters being meticulously optimized to ensure efficient EVs production (Syromiatnikova et al., 2022; Jankovic et al., 2023; Jeske et al., 2023). Dynamic culture systems, particularly hollow fiber bioreactors, CELLine bioreactors utilizing two-compartment technology, and three-dimensional cell culture bioreactors (Figure 3), have emerged as leading platforms due to their provision of extensive surface area, uniform nutrient distribution, and efficient metabolite dispersion, thereby enhancing cell density and EVs yield (Mitchell et al., 2008; Storm et al., 2016; Syromiatnikova et al., 2022; Pan et al., 2023). The integration of perfusion culture strategies with precise regulation of critical parameters, including temperature, pH, dissolved oxygen concentration, shear stress, and medium formulation, is essential for maintaining cellular viability, enhancing EVs secretion, and reducing stress-induced non-specific effluents (Kimiz-Gebologlu and Oncel, 2022).

In the quest to increase EVs yield, researchers strategically employ targeted physiological or chemical stimuli to activate cellular secretory mechanisms (Qu et al., 2023). This includes replicating hypoxic conditions, such as 1% oxygen tension, to stimulate EVs release from specific cell types; utilizing chemomodulators like cyclophosphamide to induce cellular stress and subsequently stimulate EVs synthesis; and meticulously modulating bioactive signaling molecules, such as growth factors and cytokines, to finely adjust cellular secretion processes (Debbi et al., 2022; Muniz-Garcia et al., 2022). These approaches fundamentally seek to replicate natural physiological conditions or create synthetic environments favorable for EVs production.

After reaching an optimal cell density and following specific stimulation protocols, the critical step of transitioning to serum-free or reduced-serum media, which is specifically designed for EVs enrichment, is essential for maximizing EVs accumulation (Kimiz-Gebologlu and Oncel, 2022). This strategic substitution of the medium not only reduces contamination of EVs by non-specific serum proteins, thereby maintaining EVs purity, but also has the potential to enhance EVs secretion through targeted nutrient adjustments. Sustaining the culture during this phase promotes an increase in EVs yield while simultaneously preserving the integrity of the harvested EVs.

### 3.2 Isolation of extracellular vesicles

**FIGURE 4:**
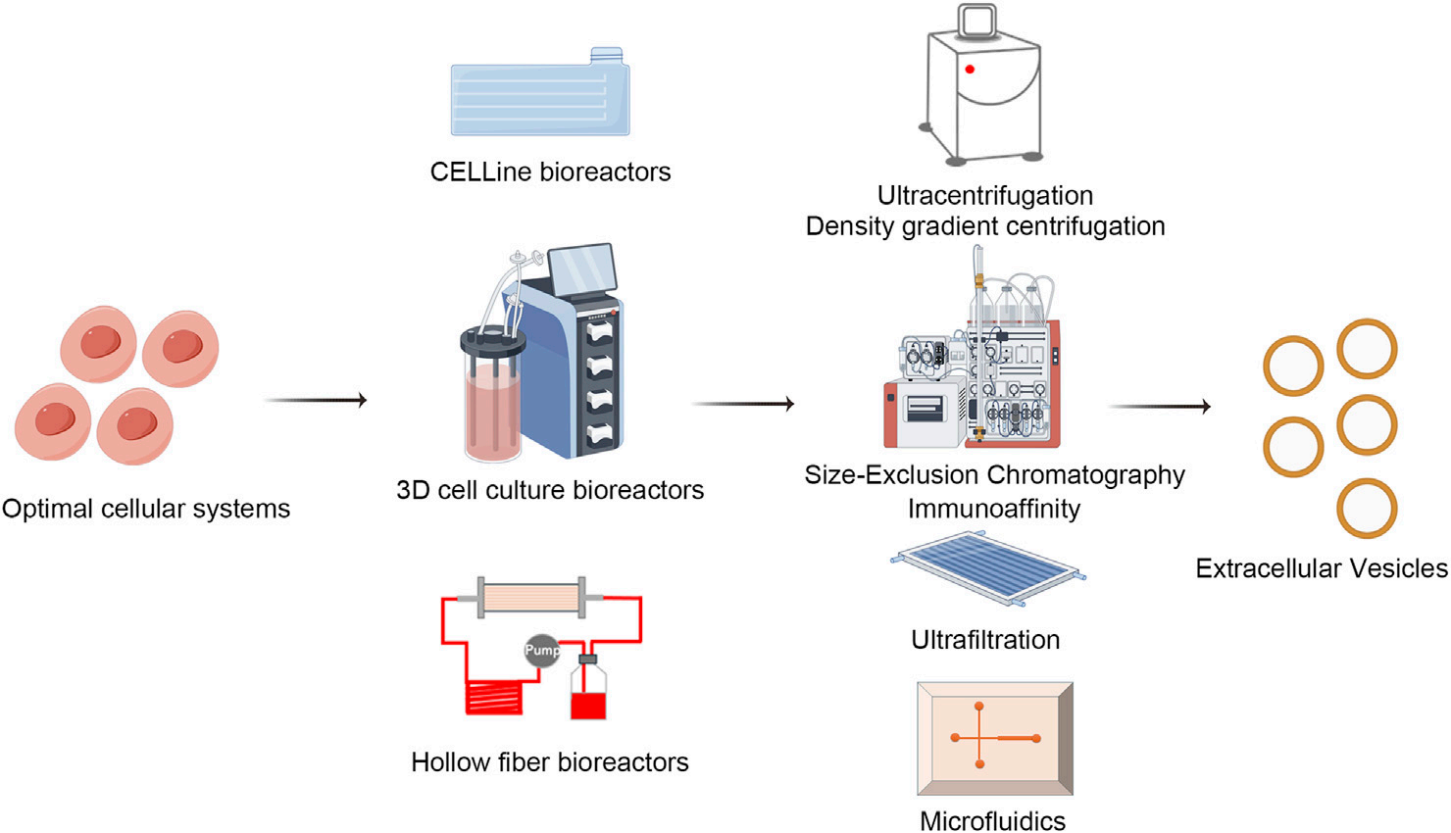
Biofabrication and isolation of EVs. EVs isolation techniques like ultracentrifugation, size exclusion chromatography, density gradient centrifugation, affinity chromatography, and microfluidics are advancing. Each method offers distinct advantages and limitations, driving progress towards achieving higher efficiency, greater purity, and minimal EVs damage, tailored to meet diverse research and clinical needs. Draw by FigDraw.

EVs purification methodologies are progressing rapidly towards achieving greater efficiency, higher purity, and minimal impact on EVs integrity (Gao et al., 2022). Prominent among these techniques are ultracentrifugation, size exclusion chromatography (SEC), density gradient centrifugation, affinity chromatography, and the emerging field of microfluidics, each providing unique advantages suited to specific applications (Figure 4) (Guan et al., 2020; Shu et al., 2021; Turner et al., 2022).

Ultracentrifugation, a traditional method, employs high centrifugal forces to primarily isolate EVs from cellular debris, proteins, and lipids. While straightforward and relatively cost-effective, its limitations include potential damage to EVs due to prolonged centrifugation, limited yields, and incomplete impurity removal, thereby restricting its utility in applications requiring high sensitivity (Gupta et al., 2018; Langevin et al., 2019; Alameldin et al., 2021; D'Acunzo et al., 2022).

Size exclusion chromatography leverages differential pore sizes in the stationary phase to separate mixture components, effectively isolating EVs due to their uniform nanometric scale. Although it is renowned for its gentle handling that preserves EVs bioactivity, it faces challenges related to low throughput and stringent requirements concerning column materials and operational conditions, especially in large-scale processing (Yang et al., 2020).

Density gradient centrifugation, through the establishment of continuous or discontinuous density gradients within a centrifuge tube, facilitates precise separation based on sedimentation coefficients, thereby producing EVs samples of higher purity. Nonetheless, its complex protocol, extended duration, dependence on specialized equipment, and requirement for technical expertise limit its widespread adoption.

Affinity chromatography exploits the specificity of biomolecular interactions, such as antibody-antigen binding, to selectively capture tagged EVs, presenting a high specificity purification strategy. With advantages of high purity and retrieval efficiency, this method is particularly well-suited for isolating EVs that express distinctive surface markers (Mathivanan et al., 2010; Ruivo et al., 2017). However, its widespread application is limited by high costs and the potential for nonspecific adsorption.

Microfluidics, through the manipulation of minute liquid volumes in microchannels, enables miniaturized, integrated, and automated EVs isolation, thereby enhancing separation accuracy and speed (Jiang et al., 2017; Mousavi et al., 2022; Zhu et al., 2022). Additionally, it allows for real-time analysis and EVs characterization, highlighting the significant potential of precision medicine and personalized therapies. Nevertheless, challenges in chip design and fabrication, as well as issues related to scalability, continue to pose obstacles.

To address the challenges associated with large-scale production of EVs, it is often necessary to employ multiple purification techniques to ensure the high quality and purity of the final product. A traditional approach to EV separation involves the integration of differential centrifugation, ultracentrifugation, and density gradient centrifugation (Jarmalaviciute et al., 2015; Deng et al., 2019; Elgamal et al., 2020; Kronstadt et al., 2023a). This combination allows for a more precise separation of EVs across various density ranges, thereby minimizing the co-precipitation of non-specific particles and other cellular components. Centrifugation should be integrated with continuous flow centrifugation equipment to enhance the separation efficiency of EVs. An alternative approach involves the combination of ultrafiltration and size exclusion chromatography (SEC) (Watson et al., 2018; Cardoso et al., 2021; Shu et al., 2021; Buntsma et al., 2022; Kronstadt et al., 2023b). Ultrafiltration facilitates the rapid removal of large molecular impurities and serves to initially concentrate the extracellular vesicle solution. Subsequently, SEC effectively eliminates small molecular impurities, including proteins and nucleic acid fragments, thereby yielding extracellular vesicle samples of higher purity. This method represents a cost-effective and efficient strategy for production. The research of Watson et al. has shown that by combining ultrafiltration and SEC, 7.7 × 10^12^ EVs can be obtained from every milliliter of medium input into the SEC column (Watson et al., 2018). With the upgrading of microfluidic technology and nanomaterials, more and more innovative solutions are emerging, which will pave the way for the large-scale production and clinical application of EVs.

### 3.3 Storage of extracellular vesicles

The large-scale production of EVs necessitates careful consideration of efficiency, cost-effectiveness, and the preservation of their biological activity and functionality during extended storage periods. The stability of EVs is affected by several factors, including temperature, the use of cryoprotectants, and the number of freeze-thaw cycles, among others (Welch et al., 2017; Budgude et al., 2021; Su et al., 2021; Wu et al., 2021; Wright et al., 2022; Levy et al., 2023; Shen, et al., 2023; Yang et al., 2024).

Under typical conditions, short-term storage is feasible at 4°C (Romanov et al., 2019; Deville et al., 2021; Su et al., 2021; Yang et al., 2024). Recent literature indicates that EVs derived from human umbilical cord mesenchymal stem cells (hUM-MSCs) exhibit no significant alterations in morphology, nucleic acid content, or biological function after 2 weeks of storage at 4°C (Su et al., 2021). However, the majority of studies suggest that the stability of EVs at 4°C is typically maintained for only up to 1 week (Maroto et al., 2017; Kanno et al., 2023; Saenz-de-Juano et al., 2024). In comparison, long-term storage at −80°C, as opposed to −20°C, has been associated with superior outcomes regarding particle concentration, nucleic acid content, morphology, and the preservation of biological functions (Gorgens et al., 2022; Wright et al., 2022). It is important to highlight that the stability of EVs is not necessarily enhanced at lower temperatures (Susa et al., 2023). Research conducted by Wu et al. has demonstrated that, in comparison to liquid nitrogen, storing EVs at −80°C results in a reduced concentration loss (Wu et al., 2021).

To mitigate structural damage to EVs caused by ice crystal formation, cryoprotectants such as sucrose, trehalose, glycerol, poloxamer 188, or bovine serum albumin (BSA) are commonly incorporated into the storage solution (Bosch et al., 2016; Neupane et al., 2021; Gelibter et al., 2022; Gorgens et al., 2022; Lyu et al., 2022; Trenkenschuh et al., 2022; Ruzycka-Ayoush et al., 2023). These protective agents play a critical role in stabilizing the membrane structure of EVs, thereby mitigating physical damage induced by low temperatures and preserving their biological functionality.

Numerous studies have demonstrated that repeated freeze-thaw cycles lead to a reduction in the quantity of EVs and nucleic acid content, while concurrently increasing the average size, aggregation rate, and diminishing biological activity (Akers et al., 2016; Dong et al., 2019; Tessier et al., 2021; Wu et al., 2021; Buntsma et al., 2022). For instance, the study by Akers JC et al. reported a 37%–43% decrease in the number of EVs following three freeze-thaw cycles. These findings underscore the essential importance of minimizing freeze-thaw cycles during the transportation and storage of EVs.

In recent years, innovative methodologies, including freeze-drying and the application of hydrogels, have been explored as potential strategies to address the limitations associated with conventional liquid-phase storage (Ju et al., 2023; Ahmadian et al., 2024). Research indicates that the use of suitable cryoprotectants enables freeze-drying to preserve the size, morphology, protein content, and biological activity of EVs(Neupane et al., 2021; Guarro et al., 2022). Furthermore, the hydrogel microneedles fabricated from hyaluronic acid are capable of preserving the structural integrity and biological activity of EVs for a duration of 6 months (Bui et al., 2022). As our comprehension of EV biology deepens and technological methodologies advance, it is anticipated that more innovative techniques will be devised to enhance the preservation of EV activity and function.

### 3.4 Characterization of extracellular vesicles

#### 3.4.1 Conventional characterization methods

Extracellular vesicles, serving as essential mediators of intercellular communication, have progressively underscored their importance in both fundamental scientific research and clinical diagnostics. Therefore, the advancement of precise and efficient methodologies for EVs characterization is imperative for elucidating their biological functions and harnessing their potential as biomarkers for various diseases. s. Currently, a diverse array of techniques is utilized to analyze EVs properties, with several widely accepted approaches including electron microscopy (Ishii et al., 2023; Li et al., 2023a; Sun et al., 2023; Che et al., 2024), atomic force microscopy (Lozano-Andres et al., 2023; Chelnokova et al., 2024; Manganelli et al., 2024), dynamic light scattering (DLS) (Bin-Bin et al., 2022; Norouzi et al., 2022; Liu et al., 2023; Mahmoudi-Aznaveh et al., 2023; Mousavi et al., 2023), nanoparticle tracking analysis (van der Pol et al., 2014; Coughlan et al., 2020; Griffiths et al., 2020; Liu et al., 2020b), Western blotting (Kowal et al., 2017; Bin-Bin et al., 2022), enzyme-linked immunosorbent assays (ELISAs) (Logozzi et al., 2020; Jin et al., 2023), and flow cytometry (Tian et al., 2020; Li et al., 2021). These methodologies collectively facilitate the elucidation of the complex roles of EVs in both health and disease.

Electron microscopy techniques, including Scanning Electron Microscopy (SEM) and Transmission Electron Microscopy (TEM), are considered the gold standard f or directly visualizing the morphology and structure of EVs. These techniques provide immediate morphological insights while requiring only minimal sample quantities (Niu et al., 2020; Ansari et al., 2024). However, the preparatory procedures for these analyses carry the risk of inducing morphological alterations, potentially compromising the accuracy of the resultant data. In contrast, Atomic Force Microscopy (AFM) enables the examination of EVs three-dimensional morphology under near-native conditions, thereby obviating the necessity for fixation and staining procedures; however, dehydration during sample preparation may induce structural modifications (Takahashi et al., 2015; Lozano-Andres et al., 2023; Chelnokova et al., 2024; Cheravi et al., 2024; Manganelli et al., 2024; Nathani et al., 2024).

For the quantification of EVs dimensions, Dynamic Light Scattering (DLS) and Nanoparticle Tracking Analysis (NTA) are prominent methodologies. DLS is especially proficient in analyzing monodispersed systems, while NTA provides the advantage of real-time measurements of particle diameter and concentration (Lyu et al., 2021; Rezakhani et al., 2021; Skrika-Alexopoulos and Mark Smales, 2023). Despite their utility, both methodologies face significant challenges in distinguishing contaminants from target EVs within complex samples. Additionally, the output of Nanoparticle Tracking Analysis (NTA) is notably susceptible to variations introduced by camera calibration parameters and other experimental variables (Coughlan et al., 2020; Liu et al., 2020b; Yahata et al., 2021; Qiao et al., 2023).

At the protein level, Western blotting facilitates both qualitative and quantitative assessments of EVs surface proteins. However, this technique is complex and time-intensive, with sensitivity limitations that are particularly pronounced in the context of biological matrices. In contrast, Enzyme-Linked Immunosorbent Assays (ELISAs) represent a critical tool for the detection of EVs surface proteins, renowned for their high specificity and quantitative capabilities. However, they also suffer from extended assay durations and susceptibility to interference (Logozzi et al., 2020; Li et al., 2022).

Ultimately, the comprehensive profiling of EVs necessitates the integrated use of various analytical techniques. This synergistic approach capitalizes on the strengths of each method to mitigate their respective limitations, thereby providing a thorough and precise understanding of EVs characteristics and their complex roles in health and disease.

#### 3.4.2 Emerging characterization methods

In recent years, the field of EVs research has experienced significant advancements in emerging characterization techniques aimed at addressing the limitations of traditional methods and improving the precision, efficiency, and depth of detection. For example, Single Molecule Array (Simoa) technology, with its ultrahigh sensitivity, facilitates the precise quantification of proteins present in EVs from minute samples, thereby enabling the early detection of disease biomarkers (Tanaka et al., 2021; Ter-Ovanesyan et al., 2021; Ter-Ovanesyan et al., 2023). Mass spectrometry imaging (MSI) is an advanced analytical technique that integrates mass spectrometry with spatial localization to elucidate the spatial distribution of complex constituents within EVs, such as proteins and lipids. This approach enhances our comprehension of EVs functionality and their involvement in intercellular communication. (Fricke et al., 2017; Huang et al., 2023).

The advent of microfluidics has profoundly influenced EVs research by enabling efficient manipulation and analysis at the microscale, facilitating rapid capture, enrichment, and high-throughput screening of EVs(Mousavi et al., 2022; Zhu et al., 2022; Mondal et al., 2023). This advancement has markedly expedited progress in both fundamental research and clinical applications of EVs. Simultaneously, the utilization of High-Resolution Mass Spectrometry (HRMS), renowned for its superior performance in mass determination and trace analysis, facilitates detailed profiling of EVs contents, thereby offering a robust tool for comprehensive molecular mapping of EVs (Jia et al., 2015; Li et al., 2023b).

Fluorescence imaging technology is integral to extracellular vesicle research, as it facilitates the direct observation of the dynamic processes and spatial distribution of extracellular vesicles through the application of specific fluorescent markers, thereby providing real-time visualization data (Panagopoulou et al., 2020; Ma et al., 2024). Additionally, single particle interference measurement technology (SP-IRIS), which operates on the principle of light wave interference, enables high-precision, non-contact measurements of individual particles (Comfort et al., 2021; Deng et al., 2022; Helin et al., 2024). By employing ExoView and Leprechaun as illustrative examples, the integration of fluorescence imaging technology with single particle interferometry obviates the need for purification (Shanthi et al., 2023; Zhyvolozhnyi et al., 2024). This approach facilitates physical characterization and protein phenotype analysis at the level of individual EVs, thereby offering a more comprehensive reflection of EVs characteristics.

Furthermore, novel CRISPR-based strategies for EVs labeling, coupled with the incorporation of deep learning and artificial intelligence in EVs image analysis, are pioneering new directions in the field (Shin et al., 2020; Jin et al., 2022). The former utilizes gene editing techniques to render EVs traceable, thereby opening new avenues for studying their *in vivo* distribution and functions. The latter leverages advanced algorithms to streamline image analysis processes, enhancing the automation and accuracy of data handling while minimizing bias introduced by subjective interpretation.

## 4 Extracellular vesicles in clinical research

### 4.1 Overview of Clinical Research

**FIGURE 5:**
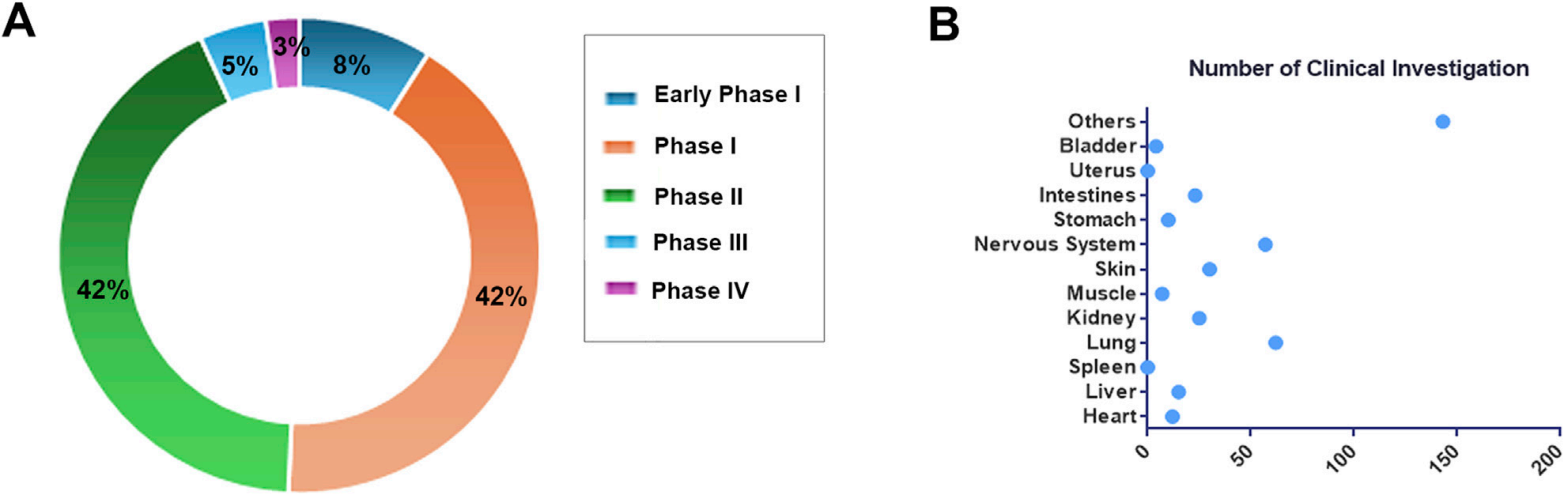
Overview of Clinical Research. **(A)** The distribution of EVs clinical studies across various clinical stages. **(B)** The research distribution of EVs in different tissues and organs.

Extracellular vesicles, serving a dual role as emerging biomarkers and promising therapeutic vectors, are currently experiencing rapid advancements in clinical research (Kok and Yu, 2020; Van Allen and Choueiri, 2021; Bai et al., 2022; Farzanehpour et al., 2023). According to statistics from the ClinicalTrials.gov database (https://classic.clinicaltrials.gov/) as of November 2024, the total number of clinical research projects related to EVs has reached 424, underscoring the substantial global attention and investment in this field (Figure 5A). Among these, 151 studies have progressed to various stages of clinical trials (Phases I-IV), comprising 76 studies in early Phase I and Phase I, 64 studies in Phase II, and 7 studies that have progressed to Phase III. Notably, the rapidly advancing Phase III clinical trials NCT05413148 and NCT05354141 are centered on mesenchymal stem cell-derived EVs. The former trial targets the treatment of retinitis pigmentosa, while the latter aims to alleviate moderate to severe Acute Respiratory Distress Syndrome (ARDS). These trials underscore significant advancements in translational EVs therapy.

We have conducted a statistical analysis of the distribution of target organs in these clinical studies (Figure 5). The majority of research efforts have concentrated on the lungs, encompassing 62 studies, particularly in relation to diseases such as chronic obstructive pulmonary disease (NCT04183530), idiopathic pulmonary fibrosis (NCT05191381), and lung cancer (NCT04529915, NCT03542253), which present challenges in early detection and effective intervention. EVs emerge as promising biomarkers and drug delivery vectors, offering novel insights (Zareba et al., 2021; Di Gioia et al., 2022; Hu et al., 2022; Ruzycka-Ayoush et al., 2023). The nervous system is the second most studied area, with 57 investigations, highlighting the intrinsic capability of EVs to traverse the blood-brain barrier. This ability is attributed to their diminutive size, lipid bilayer composition, and the presence of specific surface proteins and lipids (Matsumoto et al., 2017; Saint-Pol et al., 2020; Liang et al., 2024). For instance, in the clinical trial NCT06138210, GD-iExo-003 derived from human induced pluripotent stem cell is administered via intravenous injection for the treatment of acute ischemic stroke and is currently in Phase I of clinical trials.

Notably, cancer research is particularly prominent among EVs-related clinical studies, encompassing 166 projects, which constitutes nearly half of the total research efforts. Clinical investigations related to cancer encompass the detection of molecular markers carried by EVs, such as lncRNA and miRNA (NCT05854030, NCT03738319, NCT06015815, et al.), for purposes including early detection, disease staging, efficacy monitoring, and recurrence prediction. Currently, there are only 12 clinical studies focusing on the use of EVs in cancer treatment. Among these, notable studies include the investigation of human placental mesenchymal stem cell-derived EVs for the treatment of rectal cancer patients (NCT06536712), and the exploration of mesenchymal stromal cell-derived EVs containing KrasG12D siRNA for the treatment of metastatic pancreatic cancer patients with KrasG12D mutations (NCT03608631). As research progresses and technology advances, EVs are expected to play an increasingly pivotal role in future clinical practice, offering innovative solutions for numerous refractory diseases.

### 4.2 Mesenchymal stem cell (MSC)-derived extracellular vesicles

EVs originating from mesenchymal stem cells (MSCs) exhibit considerable potential in regenerative medicine, immune modulation, and therapeutic applications (Cao et al., 2020; Liu et al., 2020b; Qiu et al., 2020; Sun et al., 2021; Kronstadt et al., 2023a). These vesicles are characterized by robust immunoregulatory capabilities, the facilitation of tissue repair and regeneration, low immunogenicity, favorable biocompatibility, accessibility for large-scale production, and a high safety profile (Harrell et al., 2019; Wu et al., 2022; Wright et al., 2023; Arellano et al., 2024). MSC-derived EVs are particularly noteworthy for their ability to attenuate inflammatory responses through the secretion of diverse anti-inflammatory mediators and to augment immune tolerance by promoting the proliferation of regulatory T cells (Jafarinia et al., 2024; Jung et al., 2024). Furthermore, the growth factors and non-coding RNAs encapsulated within them facilitate cell proliferation and differentiation while inhibiting apoptosis, thereby enhancing wound healing and the restoration of tissue function (Lee et al., 2022; Ju et al., 2023; Wang et al., 2023). Owing to their low immunogenicity and efficient uptake by target cells, MSC-derived EVs have emerged as an optimal therapeutic delivery vehicle (Qian et al., 2024; Wang et al., 2024). Additionally, MSCs can be isolated and extensively expanded from a variety of human tissues, ensuring the large-scale production of EVs (Beeravolu et al., 2017; Jo et al., 2019; Kholodenko et al., 2019; Bunnell, 2021). MSC-derived EVs, as emerging vectors in biomedicine, have been extensively studied across a wide range of clinical contexts, spanning from ophthalmic disorders to respiratory critical illnesses, chronic degenerative conditions, and acute infectious diseases, these studies delve deeper into the central mechanisms of EVs, in intercellular communication, regenerative medicine, and disease intervention (Supplementary Table S2).

In the field of ophthalmology, EVs derived from MSCs have demonstrated efficacy in ameliorating the symptoms of dry eye syndrome (NCT05738629 and NCT04213248). Notably, in the context of post-refractive surgery dry eye syndrome, EVs preparations have exhibited both safety and effectiveness, alleviating ocular discomfort and promoting tear secretion. Furthermore, preliminary investigations into the application of MSC-derived EVs in patients with retinitis pigmentosa (RP) have yielded promising outcomes, suggesting a novel therapeutic avenue for the restoration of retinal function (NCT05413148).

Within the field of respiratory medicine, the utilization of MSCs EVs aerosol therapy in the treatment of acute respiratory distress syndrome (ARDS) and COVID-19-induced pneumonia has demonstrated significant efficacy in reducing inflammation and promoting the recovery of pulmonary function (NCT04798716 and NCT05387278). This innovative therapeutic approach offers promising interventions for severe respiratory diseases.

The therapeutic potential of MSCs EVs also extends to the management of chemotherapy-induced bone marrow suppression (NCT06245746), hepatic cirrhosis (NCT05871463), complex anal fistulas (NCT05402748), pediatric pilonidal sinus disease (NCT06391307), degenerative meniscal injuries (NCT05261360), and even the prevention of hair loss (NCT05658094). Furthermore, their involvement in neurodegenerative disorders such as ischemic stroke and Alzheimer’s disease (NCT04388982), premature ovarian insufficiency (NCT06072794), skin rejuvenation (NCT05813379), improved outcomes in coronary artery bypass grafting candidates (NCT05669144), and wound healing in diabetic ulcers underscores their multifaceted applications (NCT02138331).

In the context of tissue regeneration, EVs have demonstrated potential in treating melasma (NCT06221787), promoting corneal wound healing (NCT05243368), enhancing the efficacy of bone grafting (NCT04998058), and facilitating recovery from surgical repair of acute aortic dissection (NCT04356300), among other conditions, thereby illustrating their unique therapeutic capabilities.

EVs, as modulators of the immune system, are currently under investigation for their role in regulating inflammation in conditions such as COVID-19, autoimmune diseases, and potentially neurodegenerative disorders. This research underscores a novel Frontier in disease management through precise immune modulation.

Consequently, MSCs EVs, as versatile biomedical tools, recognized for their versatility as biomedical tools, are transforming therapeutic paradigms across a spectrum of diseases. Their comprehensive application and rigorous research highlight their pivotal role and expansive potential in contemporary medicine.

### 4.3 Extracellular vesicles in disease diagnosis

EVs, as crucial mediators of intercellular communication, are increasingly recognized for their substantial potential in clinical diagnostics, particularly within the realms of oncology, neuromuscular disorders, respiratory diseases, cardiovascular diseases, autoimmune diseases, transplant-related conditions, and metabolic disorders (Qin et al., 2020; Wan et al., 2020; Yu et al., 2022; Yu et al., 2024). Their application extends notably to early detection methodologies, such as liquid biopsy techniques. Research involves the application of microRNAs (miRNAs), long non-coding RNAs (lncRNAs), and other molecules within EVs as predictive and prognostic biomarkers across a wide range of cancers, including colorectal (NCT04523389, NCT04394572 and NCT04227886), breast (NCT05955521 and NCT01344109), gastric (NCT01779583), pancreatic (NCT04636788 and NCT04636788), lung (NCT04629079, NCT03830619, NCT04315753, NCT03542253, NCT0452991 and NCT05587114), and thyroid cancers (NCT05463107, NCT03488134, NCT04948437 and NCT02862470). Additionally, these biomarkers are utilized in the study of various conditions such as ocular myasthenia gravis (NCT05888558), epileptic respiratory dysfunction (NCT03419000), identification of acute respiratory distress syndrome (ARDS) subtypes (NCT05476029, NCT05451342), chronic kidney disease (NCT05705583), systemic lupus erythematosus (NCT04534647), renal transplant fibrosis (NCT03487861), and type 2 diabetic nephropathy (NCT06123871) (Supplementary Table S3). This underscores the versatility and potential of EVs-based diagnostics in addressing a wide array of health challenges.

EVs, in non-oncologic contexts such as cardiovascular risk stratification, post-transplantation immunosurveillance, and the management of autoimmune disorders, similarly highlight their importance. The analysis of EVs content in biofluids-such as urine (NCT04894695, NCT05270174), serum, or cerebrospinal fluid (CSF)-provides novel diagnostic insights into these complex pathologies. Innovations, particularly the development of microfluidic EVs assays, significantly enhance their application in identifying pulmonary metastases in osteosarcoma (NCT05101655). Concurrently, the exploration of CSF-derived exosomal profiles introduces a novel approach for diagnosing leptomeningeal dissemination.

Moreover, EVs, as a crucial element of liquid biopsy, demonstrate substantial potential in the early detection of prostate (NCT04720599, NCT03694483) and upper gastrointestinal malignancies (NCT06278064), as well as in assessing chemotherapeutic response in breast cancer (NCT05286684). These advancements underscore EVs as minimally invasive, high-fidelity diagnostic modalities. Collectively, advancements in EVs research are increasingly congruent with the principles of precision medicine, enhancing efforts in early disease detection, monitoring, and the customization of therapeutic regimens across a wide range of pathologies.

### 4.4 Extracellular vesicles in drug delivery

EVs, nanoscale vesicles naturally secreted by cells, have shown substantial advancements in the field of drug delivery research, highlighting their increasing importance in precision medicine and the development of novel therapeutic strategies (Jahangard et al., 2020; Han et al., 2021; Kaban et al., 2021; Ma et al., 2021; Guo et al., 2022). These minute biological carriers, characterized by their exceptional biocompatibility, low immunogenicity, and intrinsic targeting capabilities, are progressively recognized as emerging tools in drug delivery systems (Supplementary Table S4).

**FIGURE 6:**
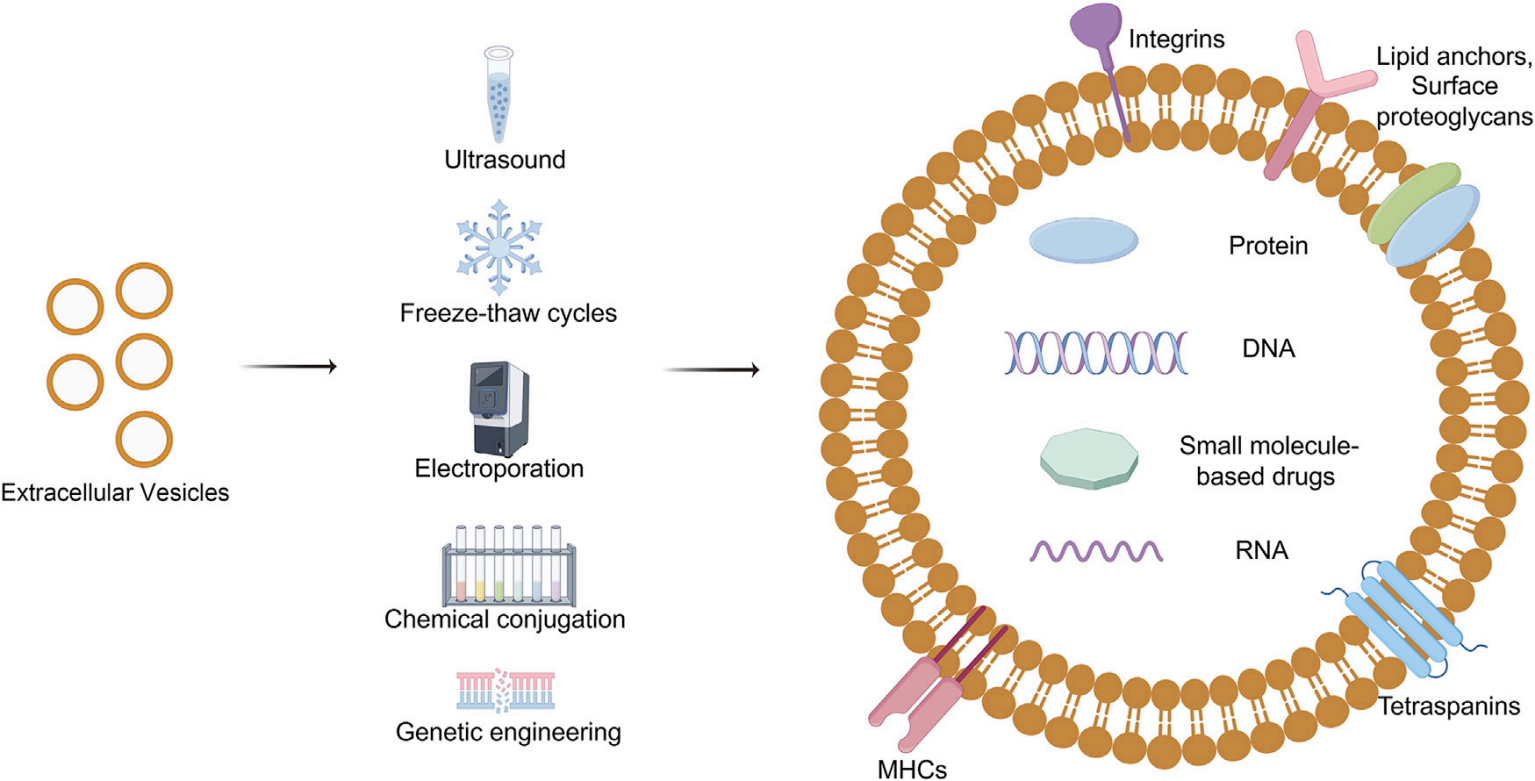
Drug loading of Extracellular vesicles. EVs loading techniques, vital for precision medicine, involve encapsulating diverse payloads like nucleic acids and drugs using methods such as direct loading, chemical modification, and cellular engineering, each presenting unique enhancements and challenges. Draw by Figdraw.

Currently, the methodologies employed for loading extracellular vesicles in clinical research encompass direct loading, chemical modification, and genetic engineering techniques (Figure 6). Direct loading involves temporarily enhancing the permeability of extracellular vesicle membranes through physical or chemical interventions, such as electroporation and freeze-thaw cycles, to facilitate the passive incorporation of drugs or nucleic acids (Liang et al., 2020; Lennaard et al., 2021; Rodriguez-Morales et al., 2021; Zhao et al., 2022). The chemical conjugation strategy entails the attachment of bioactive molecules to either the surface or interior of EVs via covalent or non-covalent interactions (Jia et al., 2018; Liu et al., 2020a; Xu et al., 2021; Zheng et al., 2022). This is achieved through techniques such as click chemistry, hydrophobic interactions, or ligand-receptor specificity to ensure precise molecular loading. Conversely, the cell engineering approach leverages advanced gene editing technologies to genetically modify cells that produce EVs, enabling them to endogenously incorporate customized molecules (Morishita et al., 2016; Bai et al., 2020; Yang et al., 2023). This approach not only preserves biological efficacy but also enhances loading efficiency.

Researchers have effectively employed EVs as vectors for the targeted delivery of specific mRNAs to recipient cells. For instance, in the treatment of familial hypercholesterolemia, EVs carrying Ldlr mRNA have been shown to successfully upregulate receptor expression, thereby presenting a novel approach to gene therapy for hereditary diseases (NCT05043181). Additionally, the utilization of plant-derived EVs to transport anti-inflammatory agents such as curcumin to affected sites introduces promising new avenues for therapeutic interventions in Inflammatory Bowel Disease (IBD) (NCT04879810).

Research into EVs-based cancer treatments is advancing rapidly. Due to their inherent homing mechanisms, EVs produced by cancer cells have been widely recognized for their targeted drug delivery capabilities in clinical trials NCT02507583 and NCT01550523, EVs secreted by malignant glioma cells were employed to deliver an IGF-1R antisense oligodeoxynucleotide. This strategy presents the potential for improved outcomes and reduced risks compared to conventional treatment modalities for tumor recurrence, such as intensified radiation and additional chemotherapy. A significant example is a Phase I clinical trial initiated in 2018 for metastatic pancreatic cancer characterized by a KrasG12D mutation. This study utilizes EVs derived from mesenchymal stromal cells to deliver KrasG12D siRNA, aiming to silence the mutated Kras gene, which is frequently associated with pancreatic cancer. As of the current date, this trial remains ongoing (NCT03608631). A recently concluded Phase II clinical trial for non-small cell lung cancer has demonstrated the safety and efficacy of EVs-mediated cancer therapy. This trial utilized EVs derived from dendritic cells to deliver metronomic cyclophosphamide, resulting in an improvement in patients’ progression-free survival (PFS) by 4 months (NCT01159288).

In summary, EVs represent an innovative platform for drug delivery, continually advancing the frontiers of medical technology and providing novel solutions to overcome traditional drug delivery challenges. As the exploration of their biological characteristics deepens and delivery techniques continue to be refined, EVs are anticipated to assume an increasingly central role in future drug design and therapeutic strategies (Qiu et al., 2020; Yang et al., 2021).

## 5 Conclusion

Extracellular vesicles, functioning as natural nanocarriers, exhibit several unique advantages that render them promising candidates for a wide range of therapeutic and diagnostic applications (Zhang et al., 2015; Ruivo et al., 2017; Kalluri and LeBleu, 2020). Their small size and lipid bilayer structure enable them to traverse biological barriers, including the blood-brain barrier, thereby making them particularly suitable for delivering therapeutics (Matsumoto et al., 2017; Saint-Pol et al., 2020; Liang et al., 2024). Furthermore, EVs exhibit inherent biocompatibility and non-immunogenic properties, thereby minimizing the risk of adverse reactions when utilized *in vivo*. They can be engineered to transport a diverse array of cargoes, such as nucleic acids, proteins, and small molecules, which can be precisely targeted to specific tissues or cell types (Watson et al., 2016; Tan et al., 2018; Yang and Teng, 2023).

Despite these promising attributes, the transition to large-scale EVs production and their successful integration into clinical practice face significant challenges as well as opportunities (Watson et al., 2018; Kimiz-Gebologlu and Oncel, 2022; Pan et al., 2023; Kink et al., 2024). The primary challenge in the upstream cell culture phase involves the screening of appropriate cell lines, achieving high-density cell proliferation, and enhancing extracellular vesicle production (Park et al., 2019; Vazquez-Rios et al., 2019; Ganesh et al., 2022). Conventional two-dimensional (2D) cultivation techniques often fall short of meeting these requirements, prompting a shift towards three-dimensional (3D) cell culture systems, including fixed bed bioreactors and microcarrier suspension cultivation (Jarmalaviciute et al., 2015; Watson et al., 2018; Deng et al., 2019; Elgamal et al., 2020; Kronstadt et al., 2023a; Kronstadt et al., 2023b). By optimizing various parameters such as the composition of the culture medium, pH levels, and temperature, it is possible to enhance cell viability and maximize the efficiency of EVs secretion (Debbi et al., 2022; Muniz-Garcia et al., 2022). The presence of extracellular vesicles in the complex culture environment presents significant challenges in achieving high-throughput and high-purity separation while preserving their integrity and biological activity. Employing a combination of traditional separation techniques, such as ultracentrifugation and density gradient centrifugation, along with ultrafiltration and size exclusion chromatography (SEC), or utilizing newly developed microfluidic technologies, can enhance separation efficiency and purity, thereby ensuring the quality and consistency of extracellular vesicles (Watson et al., 2018; Cardoso et al., 2021; Shu et al., 2021; Buntsma et al., 2022; Kronstadt et al., 2023b). Furthermore, with regard to storage, it is crucial to maintain the long-term stability and functional integrity of extracellular vesicles. The implementation of optimal low-temperature storage conditions, such as −80°C or liquid nitrogen environments, in conjunction with cryoprotectants like sucrose and trehalose, can significantly extend the shelf life of these cultures (Bosch et al., 2016; Neupane et al., 2021; Gelibter et al., 2022; Gorgens et al., 2022; Lyu et al., 2022; Trenkenschuh et al., 2022; Ruzycka-Ayoush et al., 2023). Advancements in hydrogel and freeze-drying technologies have facilitated the large-scale storage of EVs(Bui et al., 2022; Susa et al., 2023).

Furthermore, quality control is paramount in the biomanufacturing of EVs. Characterization methodologies, including nanoparticle tracking analysis (NTA), electron microscopy, and Western blotting, are employed to ensure the consistency and integrity of EVs formulations y (Ishii et al., 2023; Li et al., 2023a; Sun et al., 2023; Che et al., 2024). These assays facilitate the monitoring of critical quality attributes, such as size distribution, purity, and protein content, which are essential for therapeutic applications. However, the standardization of these methods across various laboratories and facilities continues to pose a substantial challenge. The development of new detection technologies, such as fluorescence imaging and single particle interference measurement technology (SP-IRIS), will simultaneously minimize the bias caused by subjective interpretation (Shanthi et al., 2023; Zhyvolozhnyi et al., 2024).

In summary, the accelerated advancements in EVs research are poised to fundamentally transform the medical sector, surpassing conventional diagnostic and therapeutic paradigms and fostering progress in areas such as regenerative medicine and personalized healthcare. As technologies continue to mature and scientific understanding deepens, EVs are emerging as a crucial intermediary between basic research and clinical application, guiding medicine into an era defined by remarkable precision and therapeutic effectiveness.
